# Sub-phenotypes in patients with out-of-hospital cardiac arrest who undergo extracorporeal cardiopulmonary resuscitation: a retrospective observational study from a multicenter registry

**DOI:** 10.1186/s13054-025-05575-5

**Published:** 2025-07-22

**Authors:** Masahiro Kashiura, Yuki Kishihara, Hiroyuki Tamura, Shunsuke Amagasa, Hideto Yasuda, Takashi Moriya

**Affiliations:** 1 Department of Emergency and Critical Care Medicine, Saitama Medical Center, Jichi Medical University, 1-847, Amanuma-cho, Omiya-ku, Saitama, Saitama 330-8503 Japan; 2 Division of Emergency and Transport Services, National Center for Child Health and Development, 2-10-1, Okura, Setagaya-ku, Tokyo, 157-8535 Japan; 3 Department of Clinical Research Education and Training Unit, Keio University Hospital Clinical and Translational Research Center (CTR), Shinanomachi 35, Shinjuku-ku, Tokyo, 160-8582 Japan; 4 School of Nursing, Midwifery and Social Work, UQ Centre for Clinical Research, The University of Queensland, Building 71/918, Royal Brisbane & Women’s Hospital Campus, Herston, QLD 4029 Australia; 5 School of Nursing and Midwifery, Alliance for Vascular Access Teaching and Research, Griffith University, Nathan, QLD 4111 Australia

**Keywords:** Cardiac arrest, Extracorporeal cardiopulmonary resuscitation, Latent class analysis, Sub-phenotype

## Abstract

**Background:**

Out-of-hospital cardiac arrest (OHCA) has poor survival rates, but extracorporeal cardiopulmonary resuscitation (ECPR) shows promise for selected patients, as a second line of therapy after failure of conventional CPR to obtain return of spontaneous circulation, despite implementation challenges. This study aimed to identify distinct sub-phenotypes among patients with OHCA who undergo ECPR and to investigate their association with clinical outcomes.

**Methods:**

This multi-center, retrospective, observational study used the Japanese Association for Acute Medicine OHCA registry from 83 hospitals that performed ECPR among 91 participating centers between June 2014 and December 2020. We included adult patients with OHCA who received ECPR during cardiac arrest. Three-class latent class analysis (LCA) was employed to identify sub-phenotypes based on 15 variables, including pre- and in-hospital factors. Logistic regression analysis was used to assess the association between sub-phenotypes and 30-day survival and neurological outcomes.

**Results:**

A total of 1528 patients were included. The median low-flow time was 47 min (interquartile rage: 38–58 min). The 30-day survival rate for eligible patients was 20.9%. LCA identified three distinct sub-phenotypes: Standard ECPR Group (n = 702), Delayed ECPR Group (n = 457), and Non-shockable Rhythm Group (n = 369). The variables with high discriminative power in the LCA was low-flow time, followed by pre-hospital shock delivery and initial cardiac rhythm. Thirty-day survival rates varied significantly among the sub-phenotypes (p = 0.001): Standard ECPR Group (26.9%), Delayed ECPR Group (17.1%), and Non-shockable Rhythm Group (14.1%). Favorable neurological outcomes at 30 days also differed significantly (p = 0.004), with the Standard ECPR Group showing the highest rate (12.1%). After adjusting for covariates, both the Delayed ECPR Group (adjusted OR: 0.61, 95% CI 0.44–0.82) and Non-shockable Rhythm Group (adjusted OR: 0.47, 95% CI 0.32–0.68) had significantly lower odds of 30-day survival compared to the Standard ECPR Group.

**Conclusions:**

Three clinically meaningful sub-phenotypes were identified using simple pre-hospital and in-hospital factors, with low-flow time emerging as the most critical discriminating factor. The sub-phenotypes showed significant associations with clinical outcomes and provide a practical framework for ECPR patient stratification. These findings suggest that timing optimization may be as important as rhythm characteristics for ECPR patient selection and support the development of sub-phenotype-specific treatment strategies.

**Supplementary Information:**

The online version contains supplementary material available at 10.1186/s13054-025-05575-5.

## Background

Out-of-hospital cardiac arrest (OHCA) remains a significant public health challenge, with survival rates as low as 10% [1, 2]. The prognosis for patients with OHCA is influenced by a complex interplay of factors, including patient characteristics, circumstances of the arrest, and interventions provided such as basic life support including bystander cardiopulmonary resuscitation (CPR) and early defibrillation, and advanced life support including advanced airway management and pharmacological interventions.

Recent evidence suggests that extracorporeal cardiopulmonary resuscitation (ECPR) may offer improved outcomes for select patients with OHCA [3, 4]. ECPR has emerged as a promising advanced life-support technique for refractory cardiac arrest. This approach involves the rapid initiation of veno-arterial extracorporeal membrane oxygenation (ECMO) to maintain organ perfusion while addressing the underlying cause of arrest [5, 6]. Recent randomized controlled trials and large observational studies have provided new insights into the potential benefits of ECPR, particularly in settings where it can be initiated rapidly [4, 7, 8]. Low et al. demonstrated a significant reduction in mortality (odds ratio [OR] 0.62, 95% confidence interval [CI] 0.45–0.84) [4]. This improvement appears to be particularly pronounced in settings where rapid ECPR initiation is possible, such as in a previous study where the median time from the hospital arrival to ECMO initiation was under 30 min [4, 7]. However, the overall certainty of evidence remains low to moderate, with significant heterogeneity between studies in terms of patient selection, ECPR protocols, and local healthcare system capabilities [3, 4, 7].

The implementation of ECPR presents significant logistical and resource challenges. Successful ECPR programs require a well-coordinated team, specialized equipment, and the ability to quickly identify suitable candidates [3, 6]. Moreover, the optimal timing of ECPR initiation, patient selection criteria, and post-ECPR management strategies remain subjects of ongoing research and debate [5, 9, 10]. Cost-effectiveness analyses have yielded variable results, with incremental cost-effectiveness ratios ranging widely from 12,254 to 155,739 EUR per quality-adjusted life year [3]. These factors underscore the complexity of integrating ECPR into existing resuscitation protocols and highlight the need for further research to refine its application in the management of OHCA [3, 4].

Sub-phenotype analysis of patients with cardiac arrest treated with ECPR holds significant potential for improving patient care and outcomes. This approach addresses the heterogeneity inherent in cardiac arrest populations, potentially leading to more accurate prognostic models and tailored treatment strategies [3, 4]. By identifying distinct sub-groups, it can inform the design of more targeted clinical trials, potentially increasing the power to detect intervention effects specific to certain patient profiles [5, 10]. Furthermore, sub-phenotype analysis lays the groundwork for personalized medicine approaches, where treatment decisions, including the application of ECPR, can be optimized based on a patient's specific characteristics [11, 12]. Additionally, comparing differences between sub-phenotypes may provide new insights into the pathophysiological mechanisms of post-cardiac arrest syndrome [9, 13]. Ultimately, this line of research aims to enhance clinical practice, advance scientific understanding, and improve patient outcomes in the challenging field of cardiac arrest management [6, 14].

Given the potential significance of sub-phenotype analysis in patients with cardiac arrest refractory to conventional CPR, this study aimed to identify distinct sub-phenotypes among patients who undergo ECPR and investigate their association with clinical outcomes. The application of advanced statistical methods could reveal patterns in the clinical picture that could define clinically relevant sub-groups to establish ECPR protocols and improve decision-making in clinical practice.

## Methods

### Study design and setting

This multi-center, retrospective, observational study used the OHCA registry maintained by the Japanese Association for Acute Medicine (JAAM), which collects pre- and post-hospital information regarding patients who experience OHCA in Japan [15–17]. Specifically, we extracted the data of patients who experienced OHCA and were transported to 91 hospitals in Japan between 1 June 2014 and 31 December 2020. During the study period, 91 hospitals participated in the JAAM-OHCA registry, of which 83 hospitals performed ECPR and contributed data to this analysis. In this registry, pre-hospital data are obtained from the All-Japan Utstein Registry maintained by the Fire and Disaster Management Agency [18]. In-hospital data are gathered by physicians or medical staff at each institution using an internet-based system. All the pre- and post-hospital information is registered in a web-based system. The JAAM-OHCA registry committee integrates the pre- and in-hospital data, as described previously [15]. The study was reported according to the Strengthening the Reporting of Observational Studies in Epidemiology guidelines (Table S1, Appendix A) [19].

### Study participants

We included adult patients (aged ≥ 18 years) in the JAAM-OHCA registry who underwent veno-arterial ECMO during cardiac arrest in the emergency room. ECPR implementation was performed according to each individual hospital's institutional protocols and clinical judgment, as standardized ECPR protocols were not established across participating centers during the study period. This reflects the real-world variability in ECPR practice and represents a limitation of our study. Patients with missing values for the factors used in the latent class analysis (LCA) described below were excluded. Additionally, to ensure data quality and clinical plausibility, we excluded patients with extreme outlier values in timing parameters: time from call to scene ≥ 30 min, time from scene to hospital arrival ≥ 60 min, time from hospital arrival to ECMO pump-on ≥ 60 min, and low-flow time ≥ 100 min. We did not perform missing value imputation as the number of cases with missing values was small.

### Data collection

Patient demographics and pre-hospital factors were extracted from the JAAM-OHCA registry. The data were segmented as follows: age, sex, witness status (emergency medical service personnel or others), the presence of a bystander who performed CPR, the initial cardiac rhythm, pre-hospital physician contact, pre-hospital adrenaline administration, pre-hospital airway management, pre-hospital shock delivery, the response time (the time from the call to arrival at the scene and the time from arrival at the scene to hospital arrival), and pre-hospital transient return of spontaneous circulation (ROSC). Furthermore, in-hospital factors and outcomes were extracted as follows: the cardiac rhythm on arrival, the time from hospital arrival to ECMO pump-on, the low-flow time, the etiology of the cardiac arrest (acute coronary syndrome, other cardiac disease, presumed cardiac origin, or non-cardiac origin), intra-aortic balloon pumping (IABP) implementation, percutaneous coronary intervention (PCI), targeted temperature management (TTM), and cerebral performance category (CPC) at 30 days after cardiac arrest. The low-flow time was defined as the duration from the initiation of CPR by emergency medical service personnel to ECMO pump-on. The outcome assessors were not blinded.

### Outcome measures

The primary outcome of this study was 30-day survival. The secondary outcome was favorable neurological outcome 30 days after cardiac arrest. A favorable neurological outcome was defined as a CPC score of 1 or 2. The CPC included the following five outcomes: (1) favorable cerebral recovery, (2) moderate cerebral disability, (3) severe cerebral disability, (4) coma or vegetative state, and (5) death or brain death [20].

### Statistical analysis

Descriptive statistics were calculated for all variables of interest. Continuous variables were presented as medians and interquartile ranges (IQRs), whereas categorical variables were presented as counts and percentages.

We selected the following 15 clinically important variables from the data available until the initiation of ECMO for the LCA: age, sex, witness status, the presence of a bystander who performed CPR, the initial cardiac rhythm, pre-hospital physician contact, pre-hospital adrenaline administration, pre-hospital airway management, pre-hospital shock delivery, the response time, prehospital transient ROSC, the cardiac rhythm on arrival, the time from hospital arrival to ECMO pump-on, and the low-flow time. LCA is a statistical technique that allows the identification of unobserved sub-groups within a population based on observed variables. We selected the VarSelLCM package because it can handle both categorical and continuous variables and supports variable selection to identify important clustering features [21]. LCA was initially conducted using four classes to explore the range of potential sub-phenotypes in the population. However, given that one of the four groups contained only 15 patients (less than 1% of the study population), we performed a three-class LCA to ensure statistical stability and improve clinical interpretability of the identified sub-phenotypes. The three-class model provided more balanced group sizes and clinically meaningful distinctions between sub-phenotypes. The parameters of the model were estimated using maximum likelihood estimation. This estimation enabled us to obtain the probabilities of class membership for each participant in the dataset.

To evaluate the discriminative power of the identified latent classes, the discriminative power of each variable was computed by calculating the logarithm of the ratio of the probabilities associated with the variable and its relevance to clustering. A higher variable index indicates a stronger association between the variable and the clustering process, indicating a higher discriminative power. After creating sub-phenotypes based on the model, the following analyses were performed: the Kruskal–Wallis rank sum test was used to test the continuous variables, whereas the Chi-square or Fisher’s exact tests were used to test the categorical variables.

The association between each identified sub-group and the outcomes was initially assessed using logistic regression analysis to calculate the odds ratios (ORs) and 95% CI confidence intervals (CIs) for each sub-group. The logistic regression model included the following covariates: etiology of the cardiac arrest, IABP implementation, PCI, and TTM.

Statistical analyses were conducted using R software (R Foundation for Statistical Computing, Vienna, Austria), and statistical significance was set at p < 0.05.

## Results

### Study participants

**Fig. 1:**
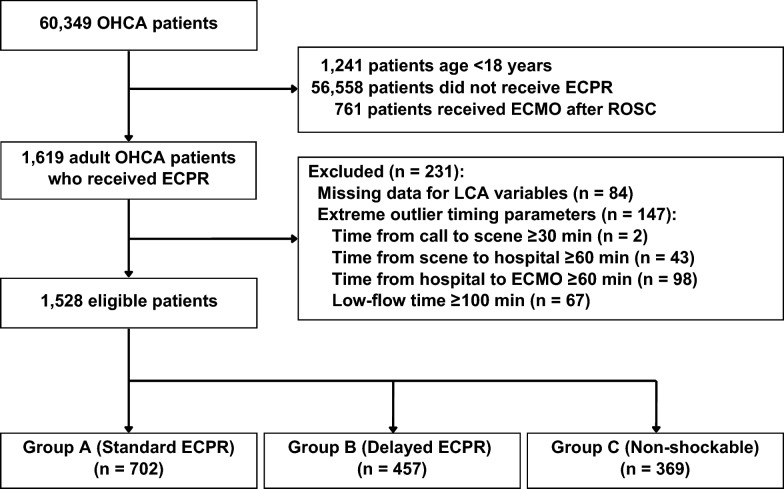
Flowchart of study participants and the number of participants in each sub-phenotype. CMO: extracorporeal membrane oxygenation; ECPR: extracorporeal cardiopulmonary resuscitation; LCA: latent class analysis; OHCA: out-of-hospital cardiac arrest; ROSC: return of spontaneous circulation

Of the 60,349 patients registered in the JAAM-OHCA Registry from 91 participating hospitals, 1,759 underwent ECPR at 83 hospitals that performed this procedure during the study period. From these ECPR cases, 231 patients were excluded: 84 patients due to missing data for one or more variables used in the LCA, and 147 patients due to extreme outlier values in timing parameters (time from call to scene ≥ 30 min [n = 2], time from scene to hospital arrival ≥ 60 min [n = 43], time from hospital arrival to ECMO pump-on ≥ 60 min [n = 98], and low-flow time ≥ 100 min [n = 67]). Finally, 1,528 adult patients with OHCA who underwent ECPR were included in the LCA (Fig. 1). The distribution of missing values among the 1,759 ECPR cases is presented in Table S2 in Appendix B.

### Overall study population characteristics

This cohort was predominantly male (83.0%), with a high rate of witnessed arrests (79.5%) and initial shockable rhythms (64.9%) (Table 1). A significant proportion received various pre-hospital interventions, including bystander CPR (49.0%), pre-hospital adrenaline administration (40.8%), and pre-hospital shock delivery (78.0%). The median time from hospital arrival to ECMO initiation was 26 min (IQR: 18–35 min), and the median low-flow time was 47 min (IQR: 38–58 min). Acute coronary syndrome was the most common etiology (45.6%). In this cohort, the overall 30-day survival rate was 20.9% (319 patients) (Table 3). Among the survivors, 144 patients (9.4% of the total population) achieved a favorable neurological outcome at 30 days.

**Table 1:** Pre-hospital and in-hospital characteristics in each sub-phenotype

	Overall (n = 1528)	Group A (standard ECPR group) (n = 702)	Group B (delayed ECPR group) (n = 457)	Group C (non-shockable rhythm group) (n = 369)	P value
Age, years [IQR]	60 [49, 69]	60 [49, 69]	59 [48, 68]	61 [50, 72]	0.019
Male, n (%)	1269 (83.0)	604 (86.0)	399 (87.3)	266 (72.1)	< 0.001
Witness, n (%)					< 0.001
EMS personnel	223 (14.6)	29 (4.1)	23 (5.0)	171 (46.3)	
Others	992 (64.9)	518 (73.8)	336 (73.5)	138 (37.4)	
Bystander CPR, n (%)	748 (49.0)	399 (56.8)	263 (57.5)	86 (23.3)	< 0.001
Initial cardiac rhythm monitored, n (%)					< 0.001
Vf/pulseless VT	991 (64.9)	612 (87.2)	379 (82.9)	0 (0.0)	
PEA	260 (17.0)	46 (6.6)	37 (8.1)	177 (48.0)	
Asystole	161 (10.5)	40 (5.7)	39 (8.5)	82 (22.2)	
Others	116 (7.6)	4 (0.6)	2 (0.4)	110 (29.8)	
Pre-hospital physician contact, n (%)	366 (24.0)	80 (11.4)	206 (45.1)	80 (21.7)	< 0.001
Pre-hospital adrenaline administration, n (%)	624 (40.8)	302 (43.0)	218 (47.7)	104 (28.2)	< 0.001
Pre-hospital advanced airway management, n (%)	897 (53.6)	177 (44.1)	6 (40.0)	607 (56.9)	0.001
Pre-hospital shock delivery, n (%)	1,192 (78.0)	701 (99.9)	444 (97.2)	47 (12.7)	< 0.001
Time from call to scene arrival, mins [IQR]	7 [6, 9]	7 [5, 8]	7 [6, 9]	7 [6, 9]	< 0.001
Time from scene arrival to hospital arrival, mins [IQR]	24 [19, 30]	22 [17, 26]	28 [22, 35]	24 [20, 31]	< 0.001
Pre-hospital transient ROSC, n (%)	100 (6.5)	43 (6.1)	40 (8.8)	17 (4.6)	0.047
Cardiac rhythm on arrival, n (%)					< 0.001
Vf/pulseless VT	772 (50.5)	446 (63.5)	285 (62.4)	41 (11.1)	
PEA	416 (27.2)	143 (20.4)	79 (17.3)	194 (52.6)	
Asystole	325 (21.3)	106 (15.1)	91 (19.9)	128 (34.7)	
Others	15 (1.0)	7 (1.0)	2 (0.4)	6 (1.6)	
Time from hospital arrival to ECMO pump-on, mins [IQR]	26 [18, 35]	20 [15, 25]	37 [32, 44]	28 [20, 36]	< 0.001
Low-flow time, mins [IQR]	47 [38, 58]	41 [35, 46]	63 [57, 71]	46 [37, 56]	< 0.001
Aetiology of cardiac arrest, n (%)					< 0.001
Acute coronary syndrome	697 (45.6)	360 (51.3)	218 (47.7)	119 (32.2)	
Other cardiac disease	348 (22.8)	183 (26.1)	120 (26.3)	45 (12.2)	
Presumed cardiac origin	247 (16.2)	110 (15.7)	78 (17.1)	59 (16.0)	
Non-cardiac origin	236 (15.4)	49 (7.0)	41 (9.0)	146 (39.6)	
Percutaneous coronary intervention, n (%)	586 (38.4)	316 (45.0)	174 (38.1)	96 (26.0)	< 0.001
Intra-aortic balloon pumping, n (%)	841 (55.0)	451 (64.2)	260 (56.9)	130 (35.2)	< 0.001
Targeted temperature management, n (%)	640 (41.9)	337 (48.0)	178 (38.9)	125 (33.9)	< 0.001

CPR: cardiopulmonary resuscitation; ECMO: extracorporeal membrane oxygenation; ECPR: extracorporeal cardiopulmonary resuscitation; EMS: emergency medical services; IQR: interquartile range; PEA: pulseless electrical activity; ROSC: return of spontaneous circulation; Vf: ventricular fibrillation; VT: ventricular tachycardia

### Survivor vs. non-survivor analysis

To complement the sub-phenotype analysis, we compared baseline characteristics between 30-day survivors (n = 319) and non-survivors (n = 1209) using the variables included in the latent class analysis (Table 2). Survivors were significantly younger (59 vs. 60 years, p = 0.039) and more likely to present with shockable rhythms (75.9% vs. 62.0%, p < 0.001). Importantly, survivors had significantly shorter timing intervals across all measured parameters: time from call to scene arrival (7 vs. 7 min, p < 0.001), time from scene arrival to hospital arrival (22 vs. 25 min, p < 0.001), time from hospital arrival to ECMO pump-on (23 vs. 27 min, p < 0.001), and crucially, shorter low-flow time (41 vs. 48 min, p < 0.001). Survivors also had higher rates of pre-hospital shock delivery (87.1% vs. 75.6%, p < 0.001) and pre-hospital transient ROSC (10.0% vs. 5.6%, p = 0.007), while having lower rates of pre-hospital adrenaline administration (34.2% vs. 42.6%, p = 0.008) and advanced airway management (47.3% vs. 55.7%, p = 0.010).

**Table 2:** Pre-hospital and in-hospital characteristics stratified by 30-day survival status

	Survived 30 days after cardiac arrest (n = 319)	Died 30 days after cardiac arrest (n = 1209)	P value
Age, years [IQR]	59 [48, 68]	60 [50, 70]	0.039
Male, n (%)	265 (83.1)	1,004 (83.0)	1.00
Witness, n (%)			0.51
EMS personnel	51 (16.0)	172 (14.2)	
Others	209 (65.5)	783 (64.8)	
Bystander CPR, n (%)	155 (48.6)	593 (49.0)	0.93
Initial cardiac rhythm monitored, n (%)			< 0.001
Vf/pulseless VT	242 (75.9)	749 (62.0)	
PEA	33 (10.3)	227 (18.8)	
Asystole	21 (6.6)	140 (11.6)	
Others	23 (7.2)	93 (7.7)	
Pre-hospital physician contact, n (%)	76 (23.8)	290 (24.0)	1.00
Pre-hospital adrenaline administration, n (%)	109 (34.2)	515 (42.6)	0.008
Pre-hospital advanced airway management, n (%)	151 (47.3)	673 (55.7)	0.010
Pre-hospital shock delivery, n (%)	278 (87.1)	914 (75.6)	< 0.001
Time from call to scene arrival, mins [IQR]	7 [5, 8]	7 [6, 9]	< 0.001
Time from scene arrival to hospital arrival, mins [IQR]	22 [17, 28]	25 [20, 31]	< 0.001
Pre-hospital transient ROSC, n (%)	32 (10.0)	68 (5.6)	0.007
Cardiac rhythm on arrival, n (%)			< 0.001
Vf/pulseless VT	220 (69.0)	552 (45.7)	
PEA	64 (20.1)	352 (29.1)	
Asystole	31 (9.7)	294 (24.3)	
Others	4 (1.3)	11 (0.9)	
Time from hospital arrival to ECMO pump-on, mins [IQR]	23 [16, 32]	27 [19, 36]	< 0.001
Low-flow time, mins [IQR]	41 [34, 53]	48 [40, 60]	< 0.001

CPR: cardiopulmonary resuscitation; ECMO: extracorporeal membrane oxygenation; EMS: emergency medical services; IQR: interquartile range; PEA: pulseless electrical activity; ROSC: return of spontaneous circulation; Vf: ventricular fibrillation; VT: ventricular tachycardia

### Latent class analysis

**Fig. 2:**
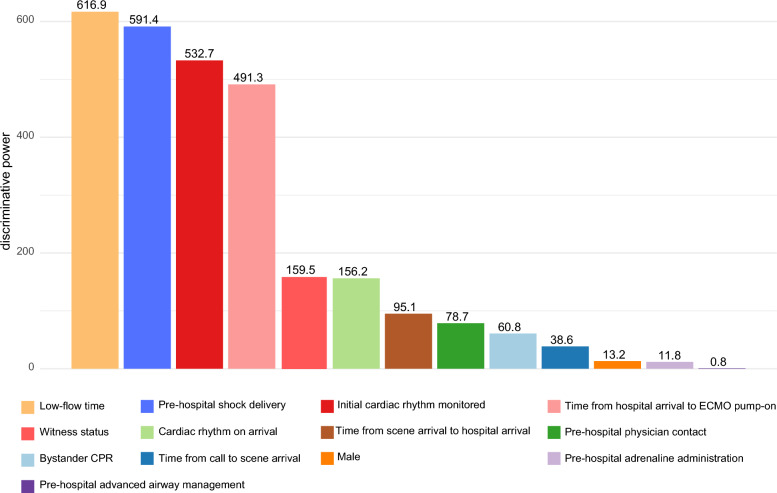
Discriminative power of each variable in descending order. CPR: cardiopulmonary resuscitation; ECMO: extracorporeal membrane oxygenation

The three-class LCA identified distinct sub-phenotypes among the 1,528 adult patients with OHCA who underwent ECPR. These sub-phenotypes were characterized as follows: Group A (n = 702), Group B (n = 457), and Group C (n = 369) (Fig. 1). Patient demographics, pre-hospital characteristics, and in-hospital factors for each sub-phenotype are presented in Table 1. The variable with the highest discriminative power in the LCA was the low-flow time, followed by pre-hospital shock delivery, and the initial cardiac rhythm monitored (Fig. 2). Other important discriminating factors included the time from hospital arrival to ECMO pump-on, witness status and cardiac rhythm on arrival.

### Sub-phenotype characteristics

Group A (n = 702) represents the standard ECPR population with optimal conditions for favorable outcomes. Patients predominantly presented with shockable rhythms (VF/VT: 87.2%), received pre-hospital shock delivery in nearly all cases (99.9%), and had the shortest low-flow time (median 41 min, IQR: 35–46). The time from hospital arrival to ECMO initiation was also the shortest (median 20 min, IQR: 15–25). This group had high rates of bystander CPR (56.8%) and witnessed arrests by others (73.8%).

Group B (n = 457) was characterized by delayed ECPR initiation despite having predominantly shockable rhythms (VF/VT: 82.9%). The distinguishing feature was prolonged low-flow time (median 63 min, IQR: 57–71) and delayed ECMO initiation (median 37 min, IQR: 32–44). Notably, this group had the highest rate of pre-hospital physician contact (45.1%), suggesting more complex pre-hospital scenarios that may have contributed to delays. The group maintained high rates of pre-hospital shock delivery (97.2%) and bystander CPR (57.5%).

Group C (n = 369) consisted entirely of patients with non-shockable rhythms (PEA: 48.0%, Asystole: 22.2%, Others: 29.8%). The group was characterized by a high proportion of EMS-witnessed arrests (46.3%) and significantly lower rates of bystander CPR (23.3%). Pre-hospital shock delivery was minimal (12.7%), reflecting the non-shockable nature of the presenting rhythms. Despite having intermediate low-flow time (median 46 min, IQR: 37–56), this group had the highest proportion of non-cardiac etiology (39.6%).

### Sub-phenotypes and clinical outcomes

Thirty-day survival rates differed significantly among the groups (p = 0.001), with the Standard ECPR Group (Group A) showing the highest survival rate (26.9%), followed by the Delayed ECPR Group (Group B, 17.1%) and the Non-shockable Rhythm Group (Group C, 14.1%) (Table 3). Thirty-day survival with favorable neurological outcomes also varied significantly among the sub-phenotypes (p = 0.004): the Standard ECPR Group had the highest rate of favorable neurological outcomes (12.1%), followed by the Non-shockable Rhythm Group (7.6%) and the Delayed ECPR Group (6.8%).

**Table 3:** Clinical outcomes of the study population in each sub-phenotype

	Overall (n = 1528)	Group A (standard ECPR group) (n = 702)	Group B (delayed ECPR group) (n = 457)	Group C (non-shockable rhythm group) (n = 369)	P value
30-day cerebral performance category, n (%)					< 0.001
1, favourable cerebral recovery	93 (6.1)	57 (8.1)	19 (4.2)	17 (4.6)	
2, moderate cerebral disability	51 (3.3)	28 (4.0)	12 (2.6)	11 (3.0)	
3, severe cerebral disability	77 (5.0)	37 (5.3)	25 (5.5)	15 (4.1)	
4, coma or vegetative state	98 (6.4)	67 (9.5)	22 (4.8)	9 (2.4)	
5, death or brain death	1209 (79.1)	513 (73.1)	379 (82.9)	317 (85.9)	
30-day survival, n (%)	319 (20.9)	189 (26.9)	78 (17.1)	52 (14.1)	0.001
30-day favourable neurological outcome, n (%)	144 (9.4)	85 (12.1)	31 (6.8)	28 (7.6)	0.004

ECPR: extracorporeal cardiopulmonary resuscitation

### Logistic regression analysis

After adjusting for covariates (etiology of cardiac arrest, IABP implementation, PCI, and TTM), logistic regression analysis revealed significant differences in outcomes among the sub-phenotypes (Table 4). For 30-day survival, compared with the Standard ECPR Group (Group A, reference), both the Delayed ECPR Group (Group B: adjusted OR: 0.61, 95% CI 0.44–0.82) and the Non-shockable Rhythm Group (Group C: adjusted OR: 0.47, 95% CI 0.32–0.68) showed significantly lower odds of survival. For 30-day neurologically favorable outcomes, the Delayed ECPR Group (adjusted OR: 0.57, 95% CI 0.36–0.87) and the Non-shockable Rhythm Group (adjusted OR: 0.64, 95% CI 0.41–0.98) both demonstrated significantly lower odds compared to the Standard ECPR Group.

**Table 4:** Odds ratios of each sub-phenotype for each outcome

	For 30-day survival		For 30-day neurologically favourable outcome	
	Crude OR (95% CI)	Adjusted OR (95% CI)	Crude OR (95% CI)	Adjusted OR (95% CI)
Group A (Standard ECPR Group)	Reference	Reference	Reference	Reference
Group B (Delayed ECPR Group)	0.56 (0.41–0.75)	0.61 (0.44–0.82)	0.53 (0.34–0.8)	0.57 (0.36–0.87)
Group C (Non-shockable Rhythm Group)	0.45 (0.32–0.62)	0.47 (0.32–0.68)	0.60 (0.38–0.92)	0.64 (0.41–0.98)

CI: confidence interval; ECPR: extracorporeal cardiopulmonary resuscitation; OR: odds ratio

## Discussion

This study identified three clinically meaningful sub-phenotypes among patients with OHCA undergoing ECPR, characterized by distinct clinical features and significantly different outcomes. These findings contribute to our understanding of ECPR patient heterogeneity and provide new insights for optimizing patient selection and treatment strategies.

### Novel identification of clinically relevant sub-phenotypes

Our study successfully identified three clinically interpretable sub-phenotypes: the Standard ECPR Group (optimal conditions), the Delayed ECPR Group (prolonged timing despite shockable rhythms), and the Non-shockable Rhythm Group (non-shockable presentations). Unlike previous studies that identified sub-phenotypes in general OHCA populations, our analysis focused specifically on the ECPR population [11, 12]. Each group can be readily identified in clinical practice: the Standard ECPR Group by optimal timing and shockable rhythms, the Delayed ECPR Group by prolonged low-flow time despite appropriate initial conditions, and the Non-shockable Rhythm Group by presenting rhythm characteristics [3, 8]. This clear clinical delineation enables practical implementation in ECPR decision-making [6, 10].

### Low-flow time and timing factors as paramount discriminating factors

A critical finding is the identification of low-flow time and ECMO pump-on timing as discriminating factors that are equal to, or potentially more important than, traditional prognostic indicators such as shockable rhythm [3–5]. While shockable rhythm has long been considered the primary determinant of ECPR candidacy, our analysis demonstrates that timing factors may be equally or more crucial for patient stratification [22, 23]. The dramatic differences in low-flow time between groups (Standard ECPR Group: 41 min vs. Delayed ECPR Group: 63 min) demonstrate that total ischemic burden is a key determinant of sub-phenotype classification [13, 24]. The 22-min difference between groups, despite similar presenting characteristics, highlights that timing optimization may be as critical as appropriate rhythm selection for achieving favorable outcomes [23].

### Complex role of pre-hospital physician contact

Our analysis revealed a complex finding regarding pre-hospital physician contact: the Delayed ECPR Group had the highest rate of pre-hospital physician contact (45.1%) yet experienced prolonged delays and intermediate outcomes. This finding requires careful interpretation in the context of recent evidence from the SAVE-J II study, which demonstrated that pre-hospital physician presence was associated with improved 30-day survival in OHCA patients undergoing ECPR (29.6% vs. 22.7%, p = 0.028) [25]. The apparent contradiction can be explained by recognizing that pre-hospital physician involvement creates a “double-edged sword” scenario: while physician involvement may provide enhanced medical expertise and decision-making capabilities, it may also contribute to treatment delays when not optimally integrated into ECPR protocols [25, 26]. Our Delayed ECPR Group represents cases where physician involvement occurred but did not translate into expedited ECPR initiation, possibly due to complex pre-hospital scenarios or communication delays. This suggests that ECPR programs should develop protocols that harness the benefits of pre-hospital physician expertise while ensuring that this involvement does not inadvertently delay definitive therapy.

### Clinical outcomes and implementation framework

The survival outcomes across our sub-phenotypes provide important benchmarks for ECPR programs [3, 4]. The Standard ECPR Group achieved a 26.9% survival rate, validating the importance of optimal patient selection and timing [8]. The 17.1% survival rate in the Delayed ECPR Group demonstrates that timing delays can significantly impact outcomes even in patients with shockable rhythms [23]. The 14.1% survival rate in the Non-shockable Rhythm Group suggests that timing optimization may partially compensate for unfavorable rhythm characteristics, supporting ECPR consideration in selected cases when rapid implementation is achievable [4, 8]. Our sub-phenotype classification provides a practical framework for ECPR decision-making that integrates both rhythm and timing considerations [6, 10]. Rather than relying solely on rhythm type, clinicians can assess patients using readily available parameters: initial rhythm, estimated low-flow time, and timing metrics.

### Implications for ECPR program development

Our findings have several implications for ECPR program development [3, 6]. The paramount importance of timing factors suggests that programs should focus on comprehensive time optimization rather than individual components [23, 27]. Quality metrics for ECPR programs should emphasize total low-flow time as a key performance indicator, with sub-phenotype-specific benchmarks: < 45 min for optimal outcomes, 45–60 min for acceptable outcomes, and > 60 min requiring urgent system improvements [13, 23]. The survival outcomes in non-shockable rhythm patients suggest that ECPR selection criteria may need expansion beyond traditional rhythm-based criteria [4, 8].

### Limitations

This study has several limitations that should be acknowledged. First, the retrospective observational design limits our ability to establish causal relationships between sub-phenotype characteristics and outcomes. Second, participating hospitals did not follow standardized ECPR protocols during the study period; instead, ECPR implementation was performed according to each individual hospital's institutional protocols and clinical judgment. This real-world variability in practice may have contributed to some of the differences observed between sub-phenotypes, particularly in timing-related factors. Third, while our sub-phenotypes demonstrate statistical and clinical significance, external validation in other healthcare systems and populations is needed. Fourth, our analysis was limited to variables available in the registry, and additional factors such as comorbidities, socioeconomic status, or detailed hospital-specific protocols may influence sub-phenotype classification. Fifth, the definition of low-flow time relied on EMS CPR initiation, which may not capture the complete ischemic period in all cases, including any period before EMS arrival. Sixth, our study was conducted in the Japanese healthcare system with its specific emergency medical services structure, and generalizability to other systems with different ECPR protocols and healthcare delivery models requires validation. Finally, a direct comparison between survivors and non-survivors according to established prognostic factors, as suggested by the reviewers, represents an important complementary analysis that should be pursued in future studies.

## Conclusions

The identification of three clinically meaningful sub-phenotypes in patients with OHCA undergoing ECPR provides a practical framework for patient stratification and outcome prediction. The paramount importance of timing factors alongside rhythm characteristics represents a significant advancement in understanding ECPR patient heterogeneity. These findings support the development of sub-phenotype-specific ECPR protocols and may contribute to more personalized and effective management of patients with cardiac arrest, ultimately improving survival and neurological outcomes.

## Supplementary Information

Supplementary Material 1.

Supplementary Material 2.

## Data Availability

Please contact the author for data requests.

## Abbreviations

OHCA: Out-of-hospital cardiac arrest
ECPR: Extracorporeal cardiopulmonary resuscitation
ECMO: Extracorporeal membrane oxygenation
CPR: Cardiopulmonary resuscitation
ROSC: Return of spontaneous circulation
LCA: Latent class analysis
JAAM: The Japanese Association for Acute Medicine
CPC: Cerebral performance category
IQR: Interquartile range
OR: Odds ratio
CI: Confidence interval
PCI: Percutaneous coronary intervention
IABP: Intra-aortic balloon pumping
TTM: Targeted temperature management
