# Supplementary material for: Sub-phenotypes in patients with out-of-hospital cardiac arrest who undergo extracorporeal cardiopulmonary resuscitation: a retrospective observational study from a multicenter registry
Source: Crit Care. 2025 Jul 22;29:316. doi: 10.1186/s13054-025-05575-5 (PMC12281804; doi:10.1186/s13054-025-05575-5)
Supplement: Supplementary file 2 — Supplementary Material 2. [file 13054_2025_5575_MOESM2_ESM.docx]

**Table S2.** Number of missing data of analyzed patients^*^

| **Variables** | **Adult OHCA patients who received ECPR (n=1,759)** |
| --- | --- |
| Age, n (%) | 0 (0.0) |
| Male, n (%) | 0 (0.0) |
| Witness, n (%) | 46 (2.6) |
| Bystander CPR, n (%) | 0 (0.0) |
| Initial cardiac rhythm monitored, n (%) | 0 (0.0) |
| Pre-hospital physician contact, n (%) | 0 (0.0) |
| Pre-hospital adrenaline administration, n (%) | 0 (0.0) |
| Pre-hospital advanced airway management, n (%) | 7 (0.4) |
| Pre-hospital shock delivery, n (%) | 0 (0.0) |
| Time from call to scene, n (%) | 1 (0.1) |
| Time from scene to hospital arrival, n (%) | 21 (1.2) |
| Pre-hospital transient ROSC, n (%) | 0 (0.0) |
| Cardiac rhythm on arrival, n (%) | 0 (0.0) |
| Time from hospital arrival to ECMO pump-on, n (%) | 12 (0.7) |
| Low-flow time, n (%) | 46 (2.6) |
| Etiology of cardiac arrest, n (%) | 0 (0.0) |
| Percutaneous coronary intervention, n (%) | 0 (0.0) |
| Intra-aortic balloon pumping, n (%) | 0 (0.0) |
| Targeted temperature management, n (%) | 0 (0.0) |
| 30-day cerebral performance category, n (%) | 0 (0.0) |
| 30-day survival, n (%) | 0 (0.0) |
| 30-day favorable neurological outcome, n (%) | 0 (0.0) |

Data are presented as number (proportion) for all variables.

CPR, cardiopulmonary resuscitation; ECMO, extracorporeal membrane oxygenation; ECPR, extracorporeal cardiopulmonary resuscitation; ROSC, return of spontaneous circulation.
